# Bacterial Quorum Sensing Molecule N-3-Oxo-Dodecanoyl-L-Homoserine Lactone Causes Direct Cytotoxicity and Reduced Cell Motility in Human Pancreatic Carcinoma Cells

**DOI:** 10.1371/journal.pone.0106480

**Published:** 2014-09-04

**Authors:** Ashwath S. Kumar, Jeffrey N. Bryan, Senthil R. Kumar

**Affiliations:** 1 Comparative Oncology and Epigenetics Laboratory, Veterinary Medicine and Surgery, University of Missouri, Columbia, Missouri, United States of America; 2 Harry S. Truman Veterans Hospital, Columbia, Missouri, United States of America; The Biodesign Institute, Arizona State University, United States of America

## Abstract

In spite of chemotherapeutic and surgical advances, pancreatic cancer continues to have a dismal prognosis. Metastasis due to tumor cell migration remains the most critical challenge in treating pancreatic cancer, and conventional chemotherapy is rarely curative. In the quest for more novel molecules to fight this disease, we tested the hypothesis that the *Pseudomonas aeruginosa* quorum sensing signal molecule N-3-oxo-dodecanoyl-L-homoserine lactone (O-DDHSL) would be cytotoxic to and reduce mobility of pancreatic carcinoma cells (Panc-1 and Aspc-1). Results showed a decrease in cell viability from apoptosis, diminished colony formation, and inhibition of migration of the evaluated pancreatic carcinoma cell lines. Also, cell viability decreased in the presence of O-DDHSL when cells were grown in matrigel basement membrane matrix. While messenger RNA for *IQGAP-1* decreased in Panc-1 and HPDE cells upon exposure to O-DDHSL, no change was observed in Aspc-1 cells. Cofilin mRNA expression was found to be increased in both HPDE and Panc-1 cells with marginal decrease in Aspc-1 cells. *RhoC*, a Rho-family GTPase involved in cell motility, increased in the presence of O-DDHSL, suggesting a possible compensatory response to alteration in other migration associated genes. Our results indicate that O-DDHSL could be an effective biomolecule in eukaryotic systems with multimodal function for essential molecular targeting in pancreatic cancer.

## Introduction

Pancreatic cancer is the fourth leading cause of cancer related mortality in the United States. According to 2013 statistics from the American Cancer Society, approximately 45,220 people (22,740 men and 22,480 women) were expected to be diagnosed with pancreatic cancer during that year. Patients are most commonly diagnosed with pancreatic cancer in an advanced stage and the disease is generally refractory to chemotherapy [1]. The most significant phenotypic change during the progression of cancer is the transition from a locally growing tumor to a metastatic phenotype. Pancreatic cancers harbor epigenetic alterations affecting phenotype, and their prevalence of these changes increases with higher grade carcinomas [2]. In spite of the discovery of gemcitabine, administered either alone or in combination with other drugs, the survival rate of 5% has remained unchanged for several decades now [3]. These facts indicate that novel compounds are required for treatment to combat this disease.

Quorum sensing (QS) is a process which enables bacterial population to communicate by means of secreted signaling molecules called autoinducers [4]. Human pathogenic bacteria such as *Pseudomonas aeruginosa (P. aeruginosa),* a Gram negative bacterium, make a range of acylhomoserine lactones (HSL) with N-acyl side chains from C4-C12 in length [5]. The *P. aeruginosa* bacteria produces a long chain HSL, N-3-oxo-dodecanoyl-L-homoserine lactone (O-DDHSL), and a short chain HSL, N-butyryl-L-homoserine (B-HSL) lactone, both of which influence the expression of virulence factors, swarming motility, and biofilm development [6]. The longer acyl side chain (eg: C12)-HSL molecules are more stable than their shorter chain counterparts (eg: C4)-HSL [7]. The shorter chain HSL can move in and out of cell membranes via free diffusion, while the longer acyl chain HSL is concentrated within the cell, possibly due to partitioning into bacterial membranes [8].

In a process called inter-kingdom signaling, bacterial QS molecules may modulate or influence the behavior of eukaryotic cells [9]. The lipophilic O-DDHSL molecule with an intact homoserine lactone ring interacts directly with phospholipids in model membrane systems and in Jurkat T-cell membranes [10]. The O-DDHSL molecule, upon entering mammalian cells [11], [12], may activate nuclear peroxisome proliferator-activated receptors (PPAR) to influence transcriptional activity and NF-κB signaling [13]. It also appears that O-DDHSL can inhibit mammalian cell proliferation and cause cell death in certain cell types, including cystic-fibrosis-airway epithelial cells [14], breast carcinoma cells [15], T-cells [16] and fibroblasts [17].

Based on existing reports that bacterial QS signals can modulate human cell behavior, we questioned whether O-DDHSL could affect pancreatic carcinoma cell phenotype and characteristics. The rationale for our studies is that pancreatic cancer patients have comparatively low survival rates and remain unresponsive to standard therapies; hence the quest for novel agents to treat pancreatic cancer is necessary. The mechanism of action of O-DDHSL in pancreatic carcinoma cells has yet to be tested. The elucidation of the mechanism of action of O-DDHSL could lead to the development of more effective analogs and novel therapeutic targets, leading to better therapeutic outcomes for pancreatic cancer patients.

The primary objective of our studies is to analyze the migration, viability and colony forming ability of pancreatic carcinoma cells *in vitro* and the effect of alteration of genes involved in these processes following O-DDHSL treatment. The central hypothesis is that O-DDHSL can modulate the genes primarily involved in pancreatic cell migration and proliferation, which include a small GTPase *Rho C* (ras homolog family member C), *cofilin* and *IQGAP-1* (IQ motif containing GTPase activating protein 1). It is expected that O-DDHSL will have multiple antitumor effects on pancreatic carcinoma cells.

## Materials and Methods

### Materials

**Figure 1:**
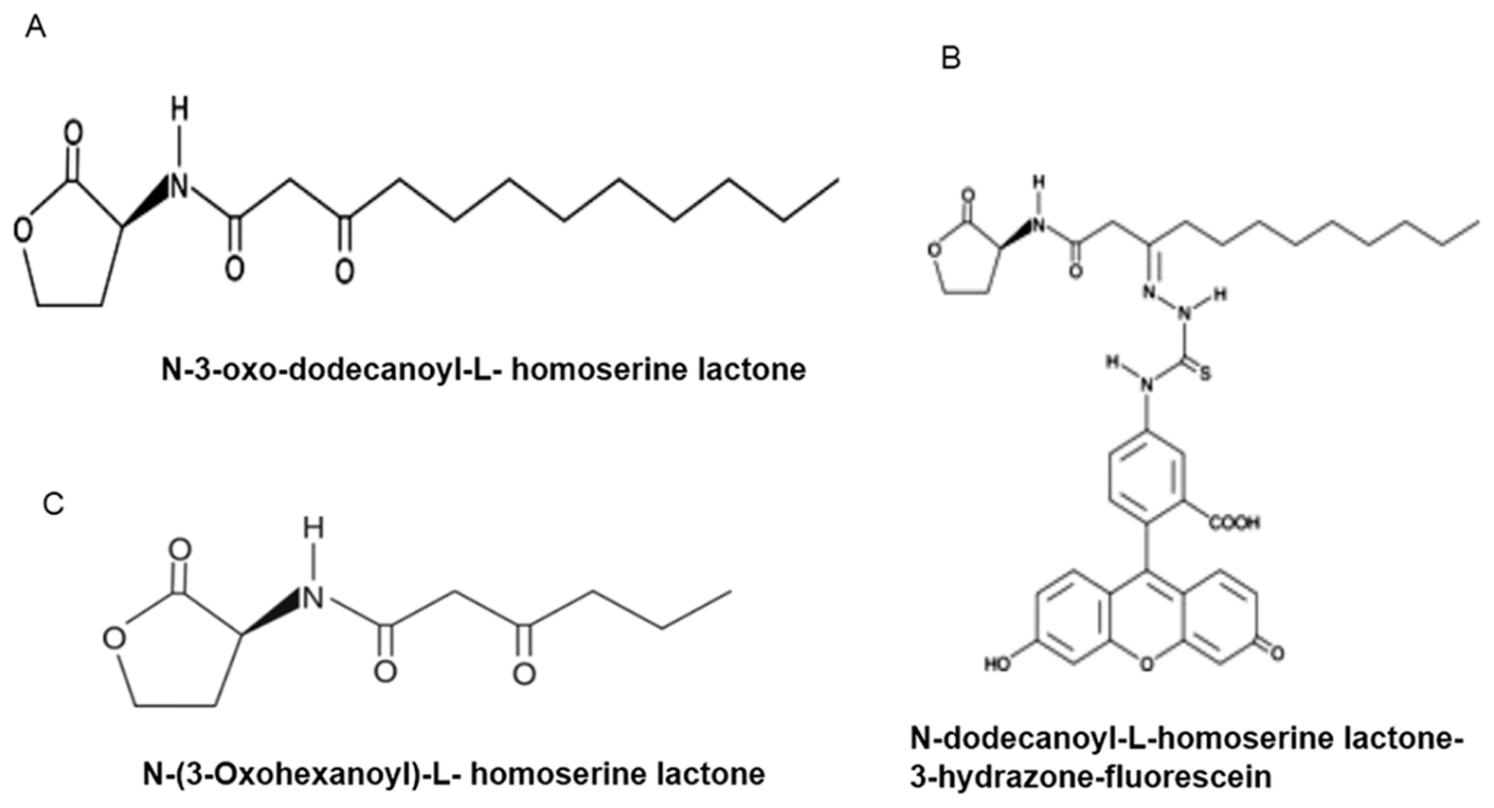
Chemical structures of homoserinelactones.

The pancreatic carcinoma cells Panc-1 and Aspc-1 were purchased from American Type Culture Collection (ATCC (CRL-1469 & CRL-1682)). Normal human pancreatic ductal epithelial cells HPDE6-C7 (HPDE) was kindly provided by Dr. Ming-Sound-Tsao, University of Toronto, Toronto, Canada (18). O-DDHSL and N-dodecanoyl-L-homoserine lactone-3-hydrazone-fluorescein (N-DD-HSL-3-HF) (Fig. 1A & B) were procured from Cayman chemicals, Ann Arbor, MI. N-(3-oxohexanoyl)-L-homoserine lactone (O-HHSL) (Fig. 1C) was purchased from Sigma Chemical Company, St Louis, MO. Antibodies for *RhoC*, *cofilin*, *IQGAP-1* and *β-actin*, including an anti-rabbit secondary antibody, were purchased from Cell Signaling Technology (Danvers, MA). All other materials were purchased from Fisher Scientific unless mentioned otherwise.

### Cell culture

Pancreatic carcinoma cells (Panc-1 and Aspc-1) were maintained in custom Roswell Park Memorial Institute (RPMI) medium containing 10% fetal bovine serum (FBS), gentamicin, L-glutamine, and pyruvate sodium. Both lines were grown at 37°C with 5% CO2. HPDE cells were grown in keratinocyte serum free media containing growth supplements at 37°C with 5% CO2.

### Cell viability assay

Cell viability assays with and without HSL molecules were performed using a WST-1 assay (Roche Applied Science) according to the manufacturer’s instructions. The HSL molecules were dissolved in 100% DMSO and further diluted in the cell growth media as required. The Panc-1, Aspc-1 and HPDE cells were seeded in 96-well plates at a density of 6×10^3^ cells per well and treated with O-DDHSL or O-HHSL (25–300 µM) or DMSO vehicle. The cells were incubated for 24 or 48 hours at 37°C and 5% CO_2_. WST-1 reagent (8 µl), a stable tetrazolium salt that is reduced by the production of NADPH on the surface of viable cells (Roche Applied Science, Indianapolis, IN), was added directly to the wells and incubated at 37°C for approximately one hour for color development. The formazan dye was quantitated by measuring the absorbance at 450 nm in a plate reader; measured absorbance directly correlated to the number of viable cells. The results are expressed as percent viable cells in both untreated and HSL treated samples. Each experiment was performed in triplicate.

### Apoptosis assay

HPDE, Panc-1 and Aspc-1 cells were seeded in a 96-well plate at a density of 6×10^3^ cells per well and treated with O-DDHSL (300 µM and 200 µM), respectively; plates were incubated for 24 hours at 37°C and 5% CO2. Apoptosis was analyzed using a high-throughput fluorescence-based assay, using quenched (z-Asp-Glu-Val-Asp)_2_-rhodamine 110 for detection of caspase 3/7 activity (Cell Technology Inc, Mountain View, CA). Following media replacement, 50 µl of apoptosis reagent for color development was added and cells were incubated for one hour according to the manufacturer’s instructions. The plate was then read at an excitation/emission wavelength of 480/520 nm. Increase in fluorescence intensity compared to untreated cells (with DMSO only), corrected for cell number indicated cell apoptosis.

The phenotypic changes of HPDE, Panc-1 and Aspc-1 upon addition of O-DDHSL (300 µM and 200 µM) were analyzed 48 h after treatment. The plates were photographed in an Olympus CK40 microscope. The pictures were pseudocolored light brown to better visualize the cells.

Cell proliferation was performed by BRDU colorimetric assay according to the manufacturer’s protocol (Roche Diagnostics, Indianapolis, IN). Briefly, (∼10^5^ cells/well) were added to 96-well plates and grown in the presence or absence of O-DDHSL (75 or 150 µM) for 48 h at 37°C. Subsequently, BRDU was added to the wells and further incubated for 2 h. After removing the culture medium the cells were fixed and denatured. Finally, anti-BRDU antibody was added to detect the BRDU incorporated in the cells. The reaction product was quantified by measuring the absorbance at 450 nm.

### Effect of O-DDHSL on cells grown in 3D culture

The 3D culture of Panc-1, Aspc-1, and HPDE cells was performed according to a model described earlier [19]. Briefly, the cells were trypsinized from a monolayer to single cell suspension and pelleted by centrifugation. Prechilled chamber slides were coated with a thin layer of Growth Factor Reduced BD Matrigel matrix (BD Biosciences, Sane Jose, and CA) maintained at 4°C. The cells were suspended in serum free (DMEM/F12) media and added to matrigel (4∶1 matrix to media) on ice. From this mixture a total of 60 µl (10^4^ cells/well) was added onto the matrigel-precoated chamber slides. After two weeks, O-DDHSL (200 µM) was added to cells in matrigel, and further incubated for 48 h at 37°C. Following incubation, Calcein AM dye (Ex/Em = 494/520 nm) (Biotium, Hayward, CA) was added to the cells and further incubated at 37°C for 2 h to check cell viability and membrane integrity. The cells were photographed using an Olympus CK40 fluorescent microscope.

### Colony Forming Assay

Cell survival was analyzed using a colony forming assay [20]. Briefly, the cells were exposed to 150 µM of O-DDHSL for 48 hours in standard cultures. After the drug treatment, the cells were washed with PBS, lifted from the culture plates and viability was tested with Trypan Blue. Live cell count was maintained the same in treated and untreated cells and transferred to 35 mm-culture dishes at a density of 2×10^4^ cells. After seven to ten days incubation at 37°C the cells were fixed with 70% methanol and stained with 0.5% crystal violet. The colonies were counted using Open CFU software [21]. Colonies, defined as groups of ≥25 cells, were identified and classified as a single colony. Clonogenic survival was expressed as a percentage relative to untreated controls and calculated as colonies per cell plated. Plating efficiency of untreated cells, based on the number of cells to number of colonies formed, was 72%.

### *In vitro* migration assay

Cell migration ability was assessed using a wound healing assay [22]. Panc-1, or Aspc-1 (2×10^4^) cells per well were seeded in 6-well plates and allowed to form a complete monolayer. The cells were treated with mitomycin-C for 2 h to block proliferation. Subsequently, a similar sized scratch was made with a sterile 200 µl pipette tip across the center of each well and immediately imaged at baseline, and 48 h, respectively, before and after treatment with O-DDHSL 150 µM (Panc-1 and HPDE) and 75 µM (Aspc-1), respectively. The image was acquired using an Olympus CK40 phase contrast microscope. The measurement of the wound gap area was performed using Image J (NIH, USA) software. An arbitrary number of one was assigned to the wound area at 0 h. The values for 48 h are relative to baseline value. Three independent experiments were performed on separate days using different cell passages.

In order to detect O-DDHSL in cells, a fluorescent analog (N-Dd-HSL-3-HF, 10 µM) was added to the live cells grown in chamber slides (40–50% confluent) and treated for about 60 min at 37°C. Subsequently, the cells were fixed with paraformaldehyde. After washing with phosphate buffered saline (PBS), the slides were photographed using a confocal laser scanning microscopy.

### cDNA synthesis and qRT-PCR analysis

Total RNA was isolated from HDPE, Panc-1 and Aspc-1 cells treated with or without O-DDHSL (150 or 75 µM for 48 h) using an RNeasy kit (Qiagen, Valencia, CA). First strand.

complimentary DNA (cDNA), obtained from the total RNA, was generated using iScript Reverse Transcription supermix (Bio-Rad, Hercules, CA). Resultant cDNA served as templates for PCR amplification with primers for *RhoC* (F-GGTCACACACCAGCACTTTA, R-TTGGAGCCTGTAGCCTTTATTC), *cofilin (*F-GAGGTGAAGCGCAAGAA, R- GGTTGCATCATAGAGGGCATAG) *IQGAP-1* (F-CCACATCCAAGACAGGCAATA, R-GGCATCCTCTGTGCTACTAAAG) and *β-actin* (F- GAAGTCCCTTGCCATCCTAAA, R- GTCTCAAGTCAGTGTACAGGTAAG). Quantitative real-time PCR (qRT-PCR) was performed with CFX Connect (Bio-Rad, Hercules, CA) using iTaq Universal SYBR Green super mix. The quantification cycle values (Cq) method (ΔΔCt) was used for the relative quantification of gene expression. Relative gene expression was calculated in the cells before and after treatment with O-DDHSL. Statistical significance was calculated using one way Analysis of Variance (ANOVA). Differences in gene expression between O-DDHSL treated and untreated samples were considered significant when P≤0.05.

### Western Blot analysis

The cell lysates from HPDE, Panc-1 and Aspc-1 was treated with M-PER mammalian protein extraction reagent (Thermo-Fisher, Rockford, IL) as per manufacturer’s instruction. Protein concentration was determined using the Bicinchoninic acid (BCA) method (Thermo-Fisher, Rockford, IL). Equal amounts of protein (∼40 µg) were separated on a 10% SDS-polyacrylamide gel and transferred to a nitrocellulose membrane (Bio-Rad, Hercules, CA). The blots were blocked at room temperature for 1 h using Tris-saline buffer (TBS) containing 0.1% Tween 20 and 10% nonfat milk. The membrane was further incubated with primary antibodies for cofilin, RhoC, IQGAP-1 or β-actin overnight in a cold chamber at 4°C in separate experiments. After being washed with TBS, the membrane was incubated with a horseradish peroxidase-labeled secondary antibody and visualized with Luminate Forte Western HRP substrate (Millipore, Billerica, MA,). The blot was imaged in a Kodak imaging station (Carestream Health, Rochester, NY).

### Statistical analysis

The data was analyzed using an unpaired two-sided Student t-test. Differences between the control and drug treated samples were considered significant when P≤0.05. Evaluation for normality was performed using the Shapiro-Wilk test.

## Results

### Cell Viability

**Figure 2:**
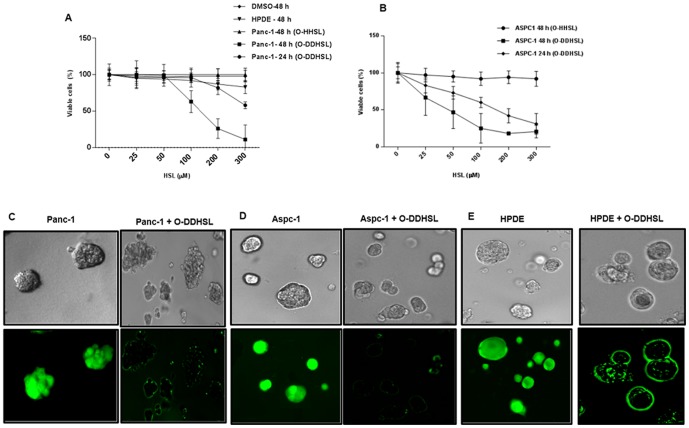
Effect of HSL on cell viability. **A**- Panc-1 (6×10^3^ cells per well) was treated with different concentrations of O-DDHSL for 24 and 48 h. Significant decrease in cell viability was observed with 200–300 µM O-DDHSL (P≤0.05) after 24 h. At 48 h, cell viability decrease was significant at O-DDHSL concentration at or above 100 µM (P≤0.02; n = 3). However, HPDE cell viability was not affected after 48 h except for a slight decrease at 300 µM O-DDHSL. No effect was observed with O-HHSL. **B** – Aspc-1 (6×10^3^ cells per well) was more sensitive to O-DDHSL exposure than Panc-1. A significant decrease (P≤0.02) was observed in viability ≥25 µM O-DDHSL after 24 or 48 h (n = 3). Cell viability was not affected by O-HHSL. In both cases, DMSO (0.02%) was used as diluent control which did not affect the cell viability *per se*. **C–E** Panc-1, Aspc-1 and HPDE cells were plated on a layer of matrigel in chamber slides in serum free DMEM/F12 media and allowed to grow for two weeks. O-DDHSL (200 µM) was added to the cells and incubated for 48 h at 37°C. Subsequently, a fluorescent dye Calcein AM was added and further incubated for 2 h for its uptake by the cells. C–E – Light microsopy pictures of cells growing on matrigel before and after addition of O-DDHSL showing morphological changes of apoptosis (top panel). Bottom panel shows the uptake of Calcein AM dye in the cells before and after treatment with O-DDHSL, showing dye uptake by viable cells and loss of cell viability resulting in the absence of dye uptake (40×).

The cell viability of Panc-1 and Aspc-1 cells was tested in the absence and presence of HSL in a time-dependent manner. The decrease in cell viability of both cell lines was concentration and time-dependent (Fig. 2 A&B). O-DDHSL inhibited (P≤0.02) Panc-1 viability at a concentration ≥100 µM when incubated for 48 h (IC_50_ = 120±5 µM) (n = 3). However, O-DDHSL had only minimal effect on HPDE cell viability. When parallel studies were conducted with O-HHSL a shorter chain HSL, no decrease in viability of Panc-1 cells was observed even at higher concentrations (300 µM). Aspc-1 cells, displayed more sensitivity (P≤0.02) to O-DDHSL in low concentrations at both 24 and 48 h (IC_50_ = 48±8 µM) (n = 3) while they also were not affected by O-HHSL. DMSO (0.02%), which was used as diluent control, did not affect the cell viability by itself.

The viability of cells grown in matrigel containing extracellular matrix components was tested in the presence of O-DDHSL. Panc-1, Aspc-1 and HPDE cells grew in matrigel as spheroids and retained the Calcein AM dye indicating viability (Figs. 2C–E). Calcein AM dye is retained by the cells if only their membranes are intact. However, the O-DDHSL treated Panc-1 and Aspc-1 cells were not able to retain the dye due to lack of viable cells (Fig. 2C&D), indicating that O-DDHSL was capable of decreasing the viability of the cells grown in an extracellular matrix environment. HPDE cell viability at the tested O-DDHSL concentration appears to be less affected compared to carcinoma cells (Fig. 2E).

### O-DDHSL induces apoptosis of pancreatic carcinoma cells

**Figure 3:**
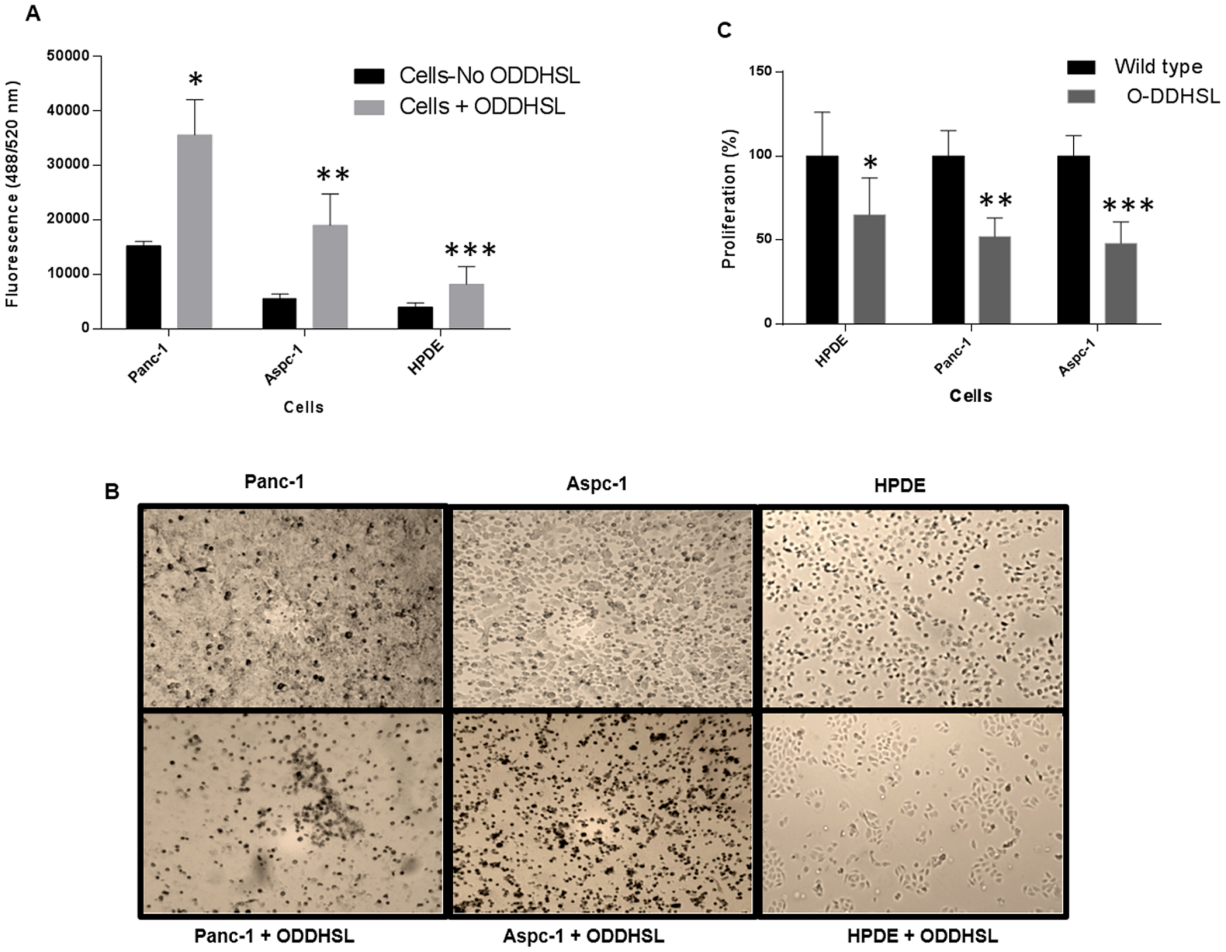
Cell apoptosis in the presence of O-DDHSL. **A**- Panc-1, Aspc-1 and HPDE-1 cells (6×10^3^ cells per well) in triplicates was treated with 300 (Panc-1 and HPDE) or 200 µM (Aspc-1) of O-DDHSL, respectively, for 24 h. Cell apoptosis was assayed using a fluorescent based caspase 3/7 activity detection kit. While apoptosis in both Panc-1 cells (P = 0.047*) and Aspc-1 cells (P≤0.02**) were significant, HPDE cell apoptosis was marginally significant (P = 0.059***) **B** – Light micrographs of Panc-1, Aspc-1 and HPDE cells before and after exposure to O-DDHSL at concentrations of 300 µM (Panc-1 and HPDE) and 200 µM (Aspc-1), respectively, for 48 h (20×). **C** – Cell proliferation of HPDE, Panc-1 and Aspc-1cells upon exposure to O-DDHSL decreased by 35% (P = 0.042*), 47% (P≤0.035 **), and 52% (P≤0.025***), respectively.

Of the mechanisms for decreased survival of pancreatic carcinoma cells exposed to O-DDHSL, apoptosis was considered most likely. Caspase 3/7 activity was detected in HPDE (P = 0.059), Panc-1(P = 0.047) and Aspc-1 cells (P≤0.02) following exposure to O-DDHSL (300 and 200 µM), after a 24 h time interval (Fig. 3A). The phenotypic changes are shown in Figure 3B. The cells become less viable and failed to proliferate. Altogether, the above studies indicate the O-DDHSL induces apoptosis in pancreatic carcinoma cells which results in decreased proliferation. Apoptosis was also observed in HPDE cells but to a lesser extent compared to carcinoma cells. Cell proliferation was independently analyzed by BRDU assay. While the proliferation was reduced by 47% (Panc-1, (P≤0.035) and 52% (Aspc-1, P≤0.025) in pancreatic carcinoma cells only about 35% decrease (P = 0.042) in proliferation was observed in HPDE cells (Fig. 3C).

### O-DDHSL treatment reduces colony forming ability and inhibits cell migration

**Figure 4:**
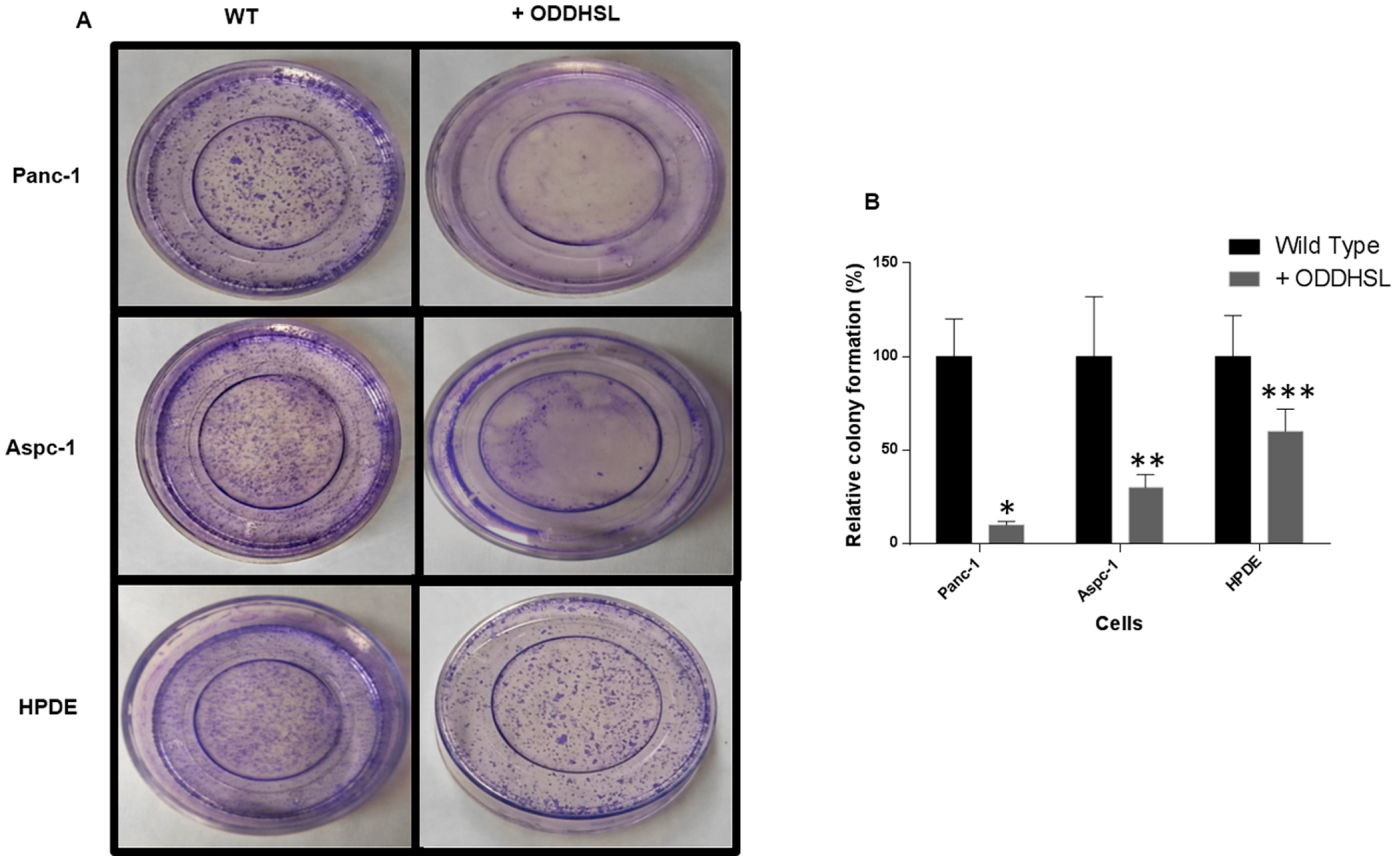
Colony formation upon treatment with O-DDHSL. **A**- Colony forming assay was performed by plating 2×10^4^ cells per plate with or without treatment of Panc-1, Aspc-1 and HPDE cells with O-DDHSL (150 µM) for 48 h. After seven to ten days incubation at 37°C the cells were fixed with 70% methanol and stained with 0.5% crystal violet. Cell colonies were counted using Open CFU software [21]. Colonies, defined as groups of ≥25 cells, were identified and classified as a single colony. **B** – Relative colony formation depicted as a percentage between O-DDHSL untreated and treated cells, Panc-1(P≤0.029*), Aspc-1(P≤0.03**) and HPDE (P≤0.05***).

The concentration of O-DDHSL was maintained slightly higher than respective IC_50_ concentration to minimize apoptosis in the following studies. In Panc-1 and HPDE cells treated with O-DDHSL (150 µM) the colony formation was reduced by 85% and 40%, respectively, compared to untreated cells (P≤0.05) (Fig. 4A &B). In Aspc-1 cells (O-DDHSL, 75 µM) the colony formation decreased 70% compared to untreated cells.

**Figure 5:**
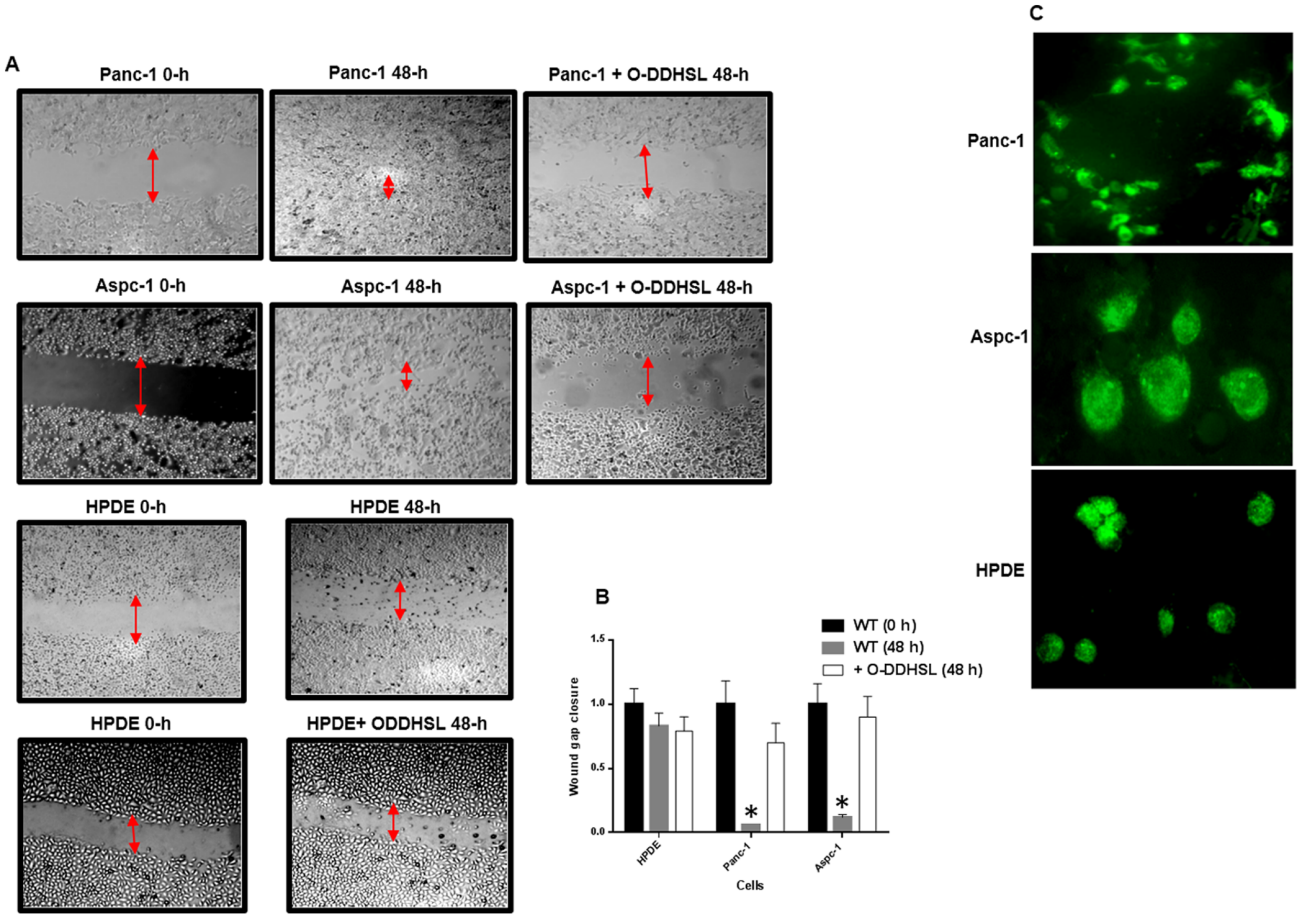
Cell migration by wound healing assay. **A**- Panc-1, Aspc-1 and HPDE cells were plated and allowed to grow as a monolayer after which a similar sized scratch was made with a sterile tip in the culture plates. The plates were photographed at baseline (0 h). A set of plates were treated with O-DDHSL (150 µM) and the gap closure was photographed after 48 h. In the presence of O-DDHSL, the migration of cells was considerably less, which resulted in non-closure of the wound gap compared to untreated cells (n = 3). Red arrows - Wound gap (20×). **B** - The wound area in the image was measured using Image J software and the baseline gap area was assigned an arbitrary number of 1. The untreated carcinoma cell wound closure values at 48 h (P = 0.027*) was significant. The O-DDHSL treated cell wound closure value at 48 h for both Pan-1 and Aspc-1 was not significant (P≥0.05). In HPDE cells, the wound closure between treated and untreated cells was almost similar. **C** – Detection of a fluorescent analog of O-DDHSL, N-Dd-HSL-3-HF, 10 µM, in different cells after 30 min at 37°C (40×).

For cell migration studies, one set of plates for each Panc-1 Aspc-1 and HPDE cells was treated with O-DDHSL (150 and 75 µM) and monitored for wound closure after 48 h. The Panc-1 cells were photographed at 0 h, and 48 h (Fig. 5A). The wound area was measured at each time point to determine the migration rate which is depicted in Figure 5B. In untreated cells, the wound area closed completely (100%) by 48 h, while in O-DDHSL treated cells the capacity of cells to migrate and close the wound gap was only 25% (Panc-1) and 10% (Aspc-1) relative to untreated cells (Fig. 5A & B). Interestingly, in the HPDE cells though some cells migrate into the wound no complete wound closure was observed. A similar result was observed in O-DDHSL treated cells. Approximately, in both cases only 15–20% wound closure was observed after 48 h.

In order to visualize the presence of O-DDHSL in the cells, a fluorescent analog, N-Dd-HSL-3-HF, 10 µM was added to the live cells grown in chamber slides. The results demonstrate uptake of fluorescent compound by the Panc-1, Aspc-1 and HPDE cells (Fig. 5C). When treated with O-HHSL, neither migration nor clonogenic ability of the cells was affected (data not shown).

### Analysis of gene expression by qRT-PCR

**Figure 6:**
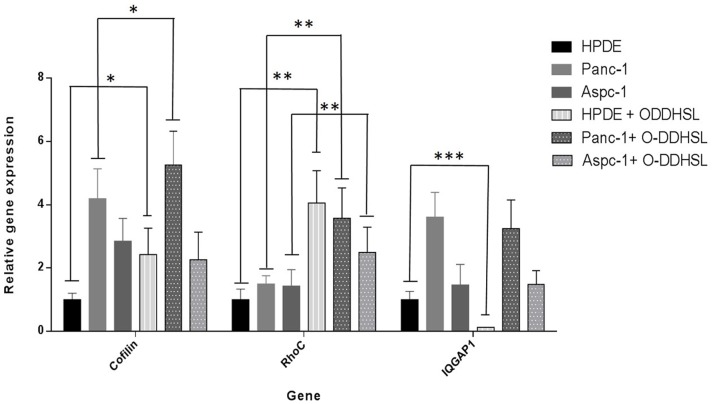
qRT-PCR analysis of genes *Rho C, Cofilin* and *IQGAP-1* in different cells. In order to analyze the changes in mRNA expression of *RhoC*, *cofilin* and *IQGAP-1* in Panc-1, Aspc-1 and HPDE cells, upon treatment with O-DDHSL (150 µM; 48 h), a qRT-PCR was performed with specific primers. *β-actin* was used as a housekeeping gene. Basal expression of all the above genes was observed in HPDE cells. *Cofilin* mRNA level increased in Panc-1 (P = 0.04*) and HPDE cells (P = 0.037*). Similarly, the RhoC mRNA levels increased in Panc-1 (P = 0.032**) and HPDE cells (P = 0.029**), respectively. The *IQGAP-1* mRNA level decreased in HPDE cells (P = 0.024***). In Aspc-1 cells, no change was observed in *IQGAP-1* mRNA, but a marginal decrease in *cofilin* and two fold (P = 0.036**) increase in *RhoC* mRNA was observed.

As metastasis is a hallmark of pancreatic cancer, we tested whether addition of O-DDHSL would alter the gene expression associated with cell migration. *RhoC*, *cofilin* and *IQGAP-1* genes are associated with cell migration and maintaining cell shape by interacting with the actin-based cytoskeleton. qRT-PCR was performed using specific gene primers for all the above mentioned genes (Fig. 6). Basal expression of these genes was found in HPDE cells. In HPDE and Panc-1 cells, increase in cofilin was two (P = 0.037) and one (P = 0.04) fold upon treatment with O-DDHSL. Similarly, the RhoC mRNA levels increased in Panc-1 by two fold (P = 0.032) and four fold in HPDE cells (P = 0.029), respectively. On the other hand, IQGAP-1 mRNA appeared to decrease one fold in Panc-1 cells and five fold (P = 0.024) in HPDE cells. In case of Aspc1 cells, while a decrease in cofilin (< one fold) and increased (two fold, P = 0.036) RhoC mRNA expression was observed no change in IQGAP-1 mRNA level upon O-DDHSL treatment between untreated and treated cells was noted.

### Western blot analysis

**Figure 7:**
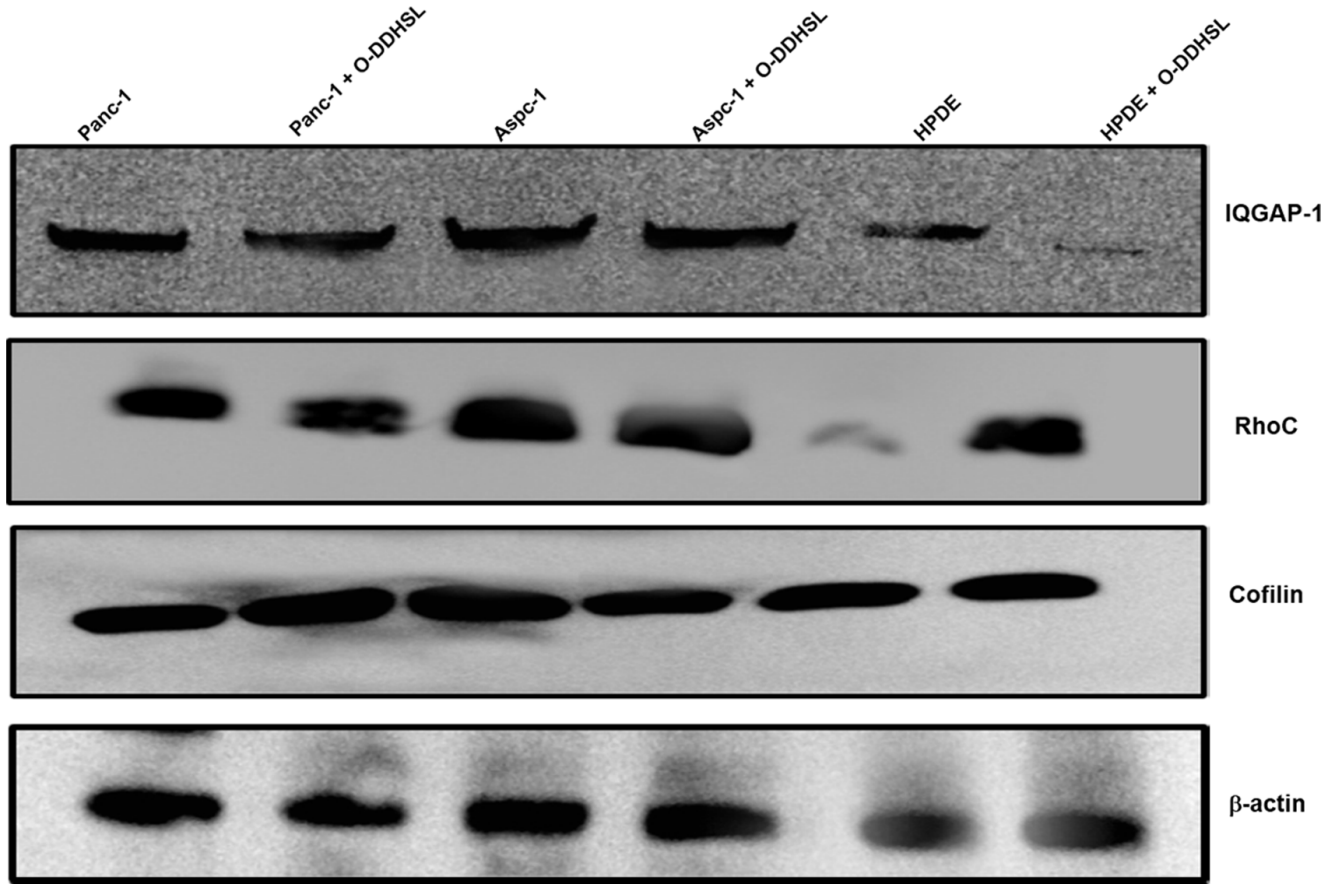
Western blot for protein expression. Equal amounts of protein (∼40 µg) was separated on a 10% SDS-polyacrylamide gel and transferred to a nitrocellulose membrane. The membrane was probed with primary antibodies for Rho-C (∼42 kDa), cofilin (∼18 kDa), *IQGAP-1 (∼197 *k*DA)* and, β-actin (∼47 kDA) followed by incubation with a HRP-conjugated secondary antibody. The bands were detected using a chemiluminescent reagent. The blot image was acquired using a Kodak imaging software.

The protein expression of *RhoC*, *cofilin* and *IQGAP-1* in Panc-1, Aspc-1 and HPDE cells was analyzed by Western blot following exposure to O-DDHSL (150 µM or 75 µM for 48 h) (Fig. 7). *β-actin* was used as a loading control. No qualitative changes in *RhoC* protein level was noted following O-DDHSL exposure in Panc-1 and Aspc-1 cells. In HPDE cells, RhoC protein expression increased compared to the wild type cells. No qualitative change in *IQGAP-1* protein expression was noted in Panc-1 and Aspc-1 cells upon O-DDHSL treatment but a qualitative decrease was observed in HPDE cells. Cofilin was moderately decreased in Aspc1- cells while a slight increase was observed in Panc-1 cells. No changes was observed with HPDE cell *cofilin* expression after treatment with O-DDHSL.

## Discussion

Bacteria communicate with eukaryotic hosts in both pathogenic and symbiotic relationships by chemical means using QS molecules. Eukaryotes can detect and react to such bacterial signals. We explored the possibility that human pancreatic tumor cells could respond to QS molecules of *P. aeruginosa* bacteria, anticipating that such signaling molecules could modulate the growth and migration characteristics as well as gene expression of the cells. We observed that the long acyl chain QS molecule O-DDHSL was effective in modulating the pancreatic carcinoma cell properties, while O-HSSL with a short chain acyl group was not as effective.

Previous studies have reported that O-DDHSL was able to decrease the cell proliferation in breast carcinoma cells and murine T-cells [15], [16]. Our studies indicate that O-DDHSL decreased the viability of pancreatic carcinoma cells in a concentration and time-dependent manner. Cells grown in 3D cultures provide a more physiologically relevant approach to the analysis of cell phenotypes and the influence of chemotherapy drugs (19). In the present study, we observed that O-DDHSL effectively decreased the viability of carcinoma cells when grown in 3D cultures relative to normal pancreatic epithelial cells. Another, shorter acyl side chain, QS molecule O-HHSL, was not effective in decreasing cell viability (Fig. 1B). This could be due the structural instability of this molecule and opening of the lactone rings in the pH and temperature of cell culture conditions [7]. The HPDE cell viability was found to be less affected by O-DDHSL (Fig. 1A). A similar behavior in the presence of O-DDHSL was reported with normal non-tumorigenic breast epithelial cells [15] CCL-185 [23], Hep-2 [23] and Caco-2 epithelial cells [24] but not cystic fibrosis airway epithelial cells [25]. The O-DDHSL concentration used in our study is within reasonable limits because it has been reported that very high levels of QS molecules can accumulate in biofilms grown *in vitro*, resulting in O-DDHSL concentrations up to 300–600 µM [17].

Apoptosis induction by O-DDHSL has been reported in a variety of cells resulting in caspase activation [15], [23]. O-DDHSL induced apoptosis in Panc-1, Aspc-1 and HPDE cells, possibly by activating caspases. The induction of apoptosis, however, was dependent on concentration and cell type. While Panc-1 and HPDE cells required higher concentration of O-DDHSL to trigger apoptosis (300 µM), Aspc-1 cells underwent apoptosis with lower concentrations (200 µM). It is possible different cell types within the same type of cancer could vary in their sensitivity for O-DDHSL.

An important characteristic of pancreatic adenocarcinoma cells is their migration and invasive properties with nearly half of patients having metastasis at the time of diagnosis. We analyzed cell migration by a wound healing assay. We found that O-DDHSL exposure inhibited the migration of both Panc-1 and Aspc-1 cells, while untreated cells were able to completely close the wound gap. Based on these results, we hypothesized that the genes involved in cell motility could be a target for O-DDHSL. It is possible that migration could be affected by O-DDHSL induced apoptosis but the concentration of the compound added was reduced to near IC_50_ values to minimize apoptosis. It was difficult to assess the effect of O-DDHSL on HPDE cells as the wound healing ability was found to be similar in untreated and cells treated with O-DDHSL. It is likely that the ability of normal pancreatic epithelial cells to close the wound gap is rather a slow process compared to carcinoma cells.

To further gain insight into the inhibition of cell migration by O-DDHSL, we focused on three important genes which are essential for cell migration including *cofilin* [26] *IQGAP-1* [27] and the small GTPase *RhoC* [28]. *Cofilin* is an important regulator of actin cytoskeleton [24] and *IQGAP-1* localizes in the leading edge of migrating cells [27], [29]. *RhoC* is a small GTPase which is an important effector of tumor cell motility [28] and is expressed in pancreatic tumors [30]. Similarly, *cofilin* is also present in pancreatic carcinoma tissues and possibly promotes its progression [31].

Upon treatment with O-DDHSL, the mRNA message of *cofilin* increased in HPDE and Panc-1 cells. The endogenous mRNA expression of *RhoC* increased in O-DDHSL treated Panc-1, Aspc-1 and HPDE cells. In HPDE cells, upon O-DDHSL treatment a decrease in IQGAP-1 was noted but the change was only marginal in Panc-1 and Aspc-1 cells. Protein expression studies indicated that in O-DDHSL treated cells no drastic changes were observed in case of IQGAP-1or RhoC in tumor cells except for HPDE cells. Altogether, O-DDHSL differentially modulates the gene expression of *cofilin, RhoC* and *IQGAP-1*, all involved in cell migration. *IQGAP-1* was reported to be targeted by O-DDHSL in Caco-2 epithelial cells affecting their migration [24]. It has also been reported that more than one form of *cofilin* exist in pancreatic cancer tissues [31]. *Cofilin-1* involved in motility and invasion is up-regulated in pancreatic carcinoma tissues while muscle *cofilin-2* was reported to be downregulated [31]. At present, it is not clear whether O-DDHSL directly interact with these molecules, however, its effect seem to vary between the cells tested in this study.

Increase in *RhoC* gene expression is intriguing, because it was reported to promote tumor metastasis in melanoma [28]. While we observed increase in *RhoC* message, no significant changes in the protein expression was observed except in HPDE cells (Fig. 7). It is unclear whether *RhoC* is subjected to any posttranscriptional regulation in the carcinoma cells [32], [33]. Alternately, we speculate that an increase in *RhoC* gene upon modulation in one or other migration inducible genes such as *cofilin* or *IQGAP-1* could be due to a compensatory pathway which reflects the plasticity of cell signaling networks [34], [35]. However, increase in *RhoC* gene expression did not promote the cell migration in our studies as observed by failure of wound closure after 48 h in the presence of O-DDHSL. Previous studies reported that compensatory responses could be identified by gene expression and phosphoproteomic changes upon single drug treatment [34]. Studies with combinations of lapatinib and Ro31-8220 in KU-7 cells, a multi-kinase inhibitor, demonstrated compensatory cross-talk between p70S6 kinase and EGF receptor pathways, and the node of convergence was identified as phosphorylated BCL2-associated agonist of cell death (BAD) [35]. Further studies are required to understand the mechanism of *RhoC* increase, as this gene has been implicated as an independent mitogen from *RhoA*, and can induce metastasis in melanoma cells [28].

In conclusion, the combined findings from our study demonstrate that QS molecule O-DDHSL decreases cell viability, promotes apoptosis by activating caspases, and inhibits the wound healing process. O-DDHSL modulates genes responsible for migration such as *RhoC*, *cofilin* and *IQGAP-1.* However, we observed that O-DDHSL also affect some of the normal epithelial cells properties similar to that of tumor cells, which could be a limiting factor when it comes to the clinical application of this molecule. The potential for O-DDHSL as a possible chemotherapeutic agent for pancreatic cancer needs further *in vivo* evaluation.
